# Contrast media extravasation following CT angiography in a patient with lower-extremity atherosclerotic occlusive disease: a case report

**DOI:** 10.3389/fmed.2026.1888394

**Published:** 2026-10-15

**Authors:** CongBing Yan, Ting Zhang, Qin Xu, Shiyuan Bi

**Affiliations:** Vascular Disease Center, Shanghai Fourth People’s Hospital Affiliated to Tongji University School of Medicine, Shanghai, China

**Keywords:** case report, contrast media extravasation, CT angiography, iodinated contrast, JinHuangSan (金黄散), skin blister

## Abstract

Contrast media extravasation (CME) is a recognized complication of intravenous iodinated contrast administration during computed tomography (CT) examinations, with a reported incidence rate ranging between 0.1% and 1.2%. Although most extravasation injuries are mild and self-limiting, a small proportion of them progress to skin blistering, tissue necrosis, or compartment syndrome. Blister formation, in particular, is an uncommon manifestation observed predominantly in moderate-to-severe extravasation. Herein, we report a case of iobitridol extravasation that occurred during lower-extremity CT angiography in a 68-year-old female patient with lower-extremity atherosclerotic occlusive disease. Approximately 5 h after the procedure, multiple fluid-filled skin blisters developed at the forearm injection site. A combined external regimen-topical Hirudoid (mucopolysaccharide polysulfate) cream, intermittent ice compresses, and Jinhuang San (Golden Yellow Powder) mixed with green-tea water as a wet poultice was applied immediately. The blisters were absorbed by the following day without skin breakdown, and complete resolution was achieved within 48 h. To our knowledge, this is the first report describing this specific three-component topical combination for iobitridol extravasation presenting with blistering. This case highlights the importance of early recognition and structured topical management of CME and suggests—at the hypothesis-generating level—that integrating a traditional Chinese medicine external therapy with conventional topical agents may be a useful option for such injuries. Prospective, controlled studies are required to confirm these observations.

## 1 Introduction

Contrast-enhanced computed tomography (CECT) is widely used in clinical practice to improve visualization of anatomical structures and pathological changes. Intravenous administration of iodinated contrast media, frequently delivered by power injectors at high flow rates, is essential for vascular imaging, including CT angiography (CTA) (1). Contrast media extravasation (CME)—the accidental leakage of a contrast agent from a vessel into the surrounding soft tissue—is a well-recognized complication of this procedure. Large registry and cohort data indicate an incidence rate of approximately 0.1%–1.2% of all contrast-enhanced CT examinations; in a recent analysis of 378,082 intravenous contrast-enhanced CT scans, it was found that the frequency remained within this low range, yet the absolute number of events is substantial given the global volume of CECT that is performed (2, 3).

The clinical manifestations of CME range from mild swelling, erythema, and localized pain to severe tissue injury, including skin blistering, ulceration, necrosis, and compartment syndrome (4). The severity of injury depends on multiple factors, including the volume of extravasated contrast, the osmolality and viscosity of the agent, the injection rate, and the anatomical location (5). Non-ionic low-osmolar contrast media, although considerably safer than the earlier ionic high-osmolar agents, still carry a risk of tissue damage, particularly when large volumes extravasate (6). Importantly, blister formation is an uncommon presentation that generally reflects moderate-to-severe injury. In a national data registry analysis of 1,085 contrast extravasation events, 94.6% of injuries were classified as mild, 4.7% as moderate, and 0.8% as severe (8). In a separate observational cohort with detailed outcome reporting, 29 of 32 patients with moderate extravasation developed skin blisters (mean recovery 5 days, range 2–12), and all 5 patients with severe extravasation developed blisters (mean recovery 23 days, range 17–30) (5). Thus, although CME itself is a recognized and relatively common complication, blister formation represents an uncommon and more severe end of its clinical spectrum and warrants specific attention.

Current guidelines from the American College of Radiology (ACR) and the European Society of Urogenital Radiology (ESUR) recommend conservative management—cessation of injection, limb elevation, local compresses, and close monitoring-as first-line treatment for most cases (1, 4). Topical mucopolysaccharide polysulfate (Hirudoid) (MPS) is commonly used to reduce local inflammation and promote tissue repair (4, 5). In Chinese nursing practice, traditional Chinese medicine (TCM) external therapies, notably Jinhuang San (Golden Yellow Powder, also known as Ru-Yi-Jin-Huang-San), have been reported to be effective for drug extravasation–induced skin injury: in a clinical study, it was found that early wet compresses with this formula shortened mean healing time to 6.44 days, and an experimental iohexol extravasation model demonstrated a significant reduction of swelling area and inflammatory infiltration at 24 and 48 h, respectively (9, 10).

Despite these individual lines of evidence, the combined use of Hirudoid, ice compress, and Jinhuang San tea-water poultice for CME presenting with skin blistering has, to our knowledge, not been previously reported, and no published case has specifically described iobitridol extravasation with blistering managed by such an integrated regimen. We therefore report this case, prepared in accordance with the CARE (CAse REport) guidelines, to document the clinical course and management in detail, to discuss the plausible mechanisms of injury and treatment, and to compare our case with similar published reports.

## 2 Case report

### 2.1 Patient history and presentation

A 68-year-old female patient was admitted to the Department of Vascular Surgery with a 7-year history of left lower-extremity pain. The pain had begun without an obvious precipitating cause, was severe and intolerable, was aggravated by standing or dependent positioning, and was partially relieved by lying flat or elevating the affected limb. She had not sought medical attention during this period. One year before admission, the symptoms worsened and were accompanied by a marked sensation of coldness in the left calf and foot. She was admitted through the outpatient clinic with a diagnosis of “lower-extremity atherosclerotic occlusive disease.”

The patient’s past medical history was notable for grade 3 hypertension (very-high-risk category). Her regular medications included clopidogrel 75 mg once daily, atorvastatin 20 mg every night, nifedipine controlled-release 30 mg once daily, and telmisartan 40 mg once daily. Baseline vascular assessment showed an ankle–brachial index (ABI) of 0.9 (20 January 2026). Renal function was preserved: the estimated glomerular filtration rate (eGFR) was 125.48 mL/min/1.73 m^2^ on 20 January 2026, and 136.08 mL/min/1.73 m^2^ on 1 February 2026.

### 2.2 CTA procedure and extravasation event

On 19 January 2026, at 17:00, the patient underwent a lower-extremity CTA. A non-ionic low-osmolar contrast agent, iobitridol injection (350 mgI/mL), was administered using a power injector through a 20-G peripheral intravenous cannula placed in a forearm vein, at a flow rate of 3.5–4.0 mL/s. During the process of administration of the injection, the patient complained of distending pain at the puncture site [Numeric Rating Scale (NRS) score 6/10], following which the injection was terminated immediately; according to the injector record, a total of 80 mL had been delivered. The cannula was removed, a new puncture site was selected, and the examination was restarted and completed. After the patient’s return to the ward, bedside point-of-care ultrasound estimated the extravasated volume at approximately 5 mL.

The completed CTA demonstrated severe aortoiliac atherosclerotic disease: atherosclerosis of the abdominal aorta with severe luminal stenosis and focal distal occlusion; mixed plaques in both common iliac arteries with severe stenosis; complete occlusion of the left internal iliac artery and moderate-to-severe stenosis of the right internal iliac artery (mixed plaques); severe stenosis with focal occlusion of the left external iliac artery (mixed plaque); and severe stenosis of the right external iliac artery (soft plaque).

On return to the ward, the patient reported pain around the puncture site without obvious swelling. Hirudoid (mucopolysaccharide polysulfate) cream was applied topically to the affected area as a thin layer.

### 2.3 Clinical course, management, and outcome

At 22:00 on the same day (approximately 5 h after the extravasation), routine nursing rounds identified three to five tense, fluid-filled blisters at the injection site on the forearm (Figure 1), with an overall affected skin area of approximately 3 cm × 5 cm on a background of localized erythema and mild swelling; the NRS pain score at that time was 4/10. Capillary refill, distal sensation, and passive movement of the fingers were assessed and remained intact, with no clinical evidence of compartment syndrome at any point during the course; the skin was reassessed hourly by the nursing team.

**Figure 1:**
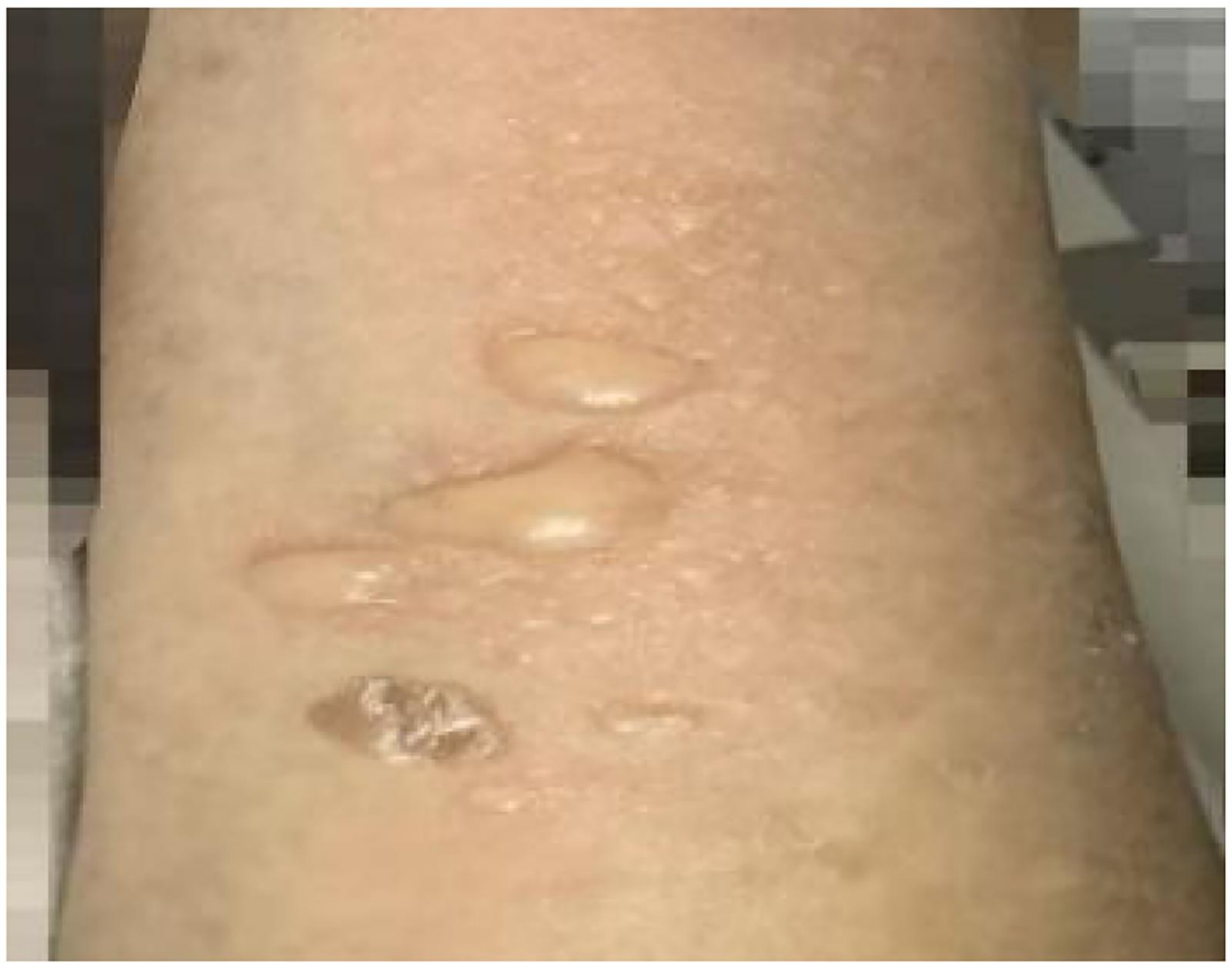
Multiple tense, fluid-filled blisters at the forearm injection site approximately 5 h after contrast media (iobitridol) extravasation.

Immediate escalation of topical management was initiated: (i) intermittent ice-bag compresses applied to the affected area for 15–20 min every 2–4 h, with a cloth barrier to protect the blistered skin; (ii) Jinhuang San (Golden Yellow Powder) mixed with cooled green-tea water at a 1:1 ratio to form a paste, applied as an even layer approximately 2–3 mm thick over the affected area, covered with sterile gauze, and changed every 12 h (two applications in total) (Figure 2); and (iii) continued Hirudoid cream to the surrounding intact skin between dressings, with the limb elevated above heart level. The blister roofs were kept intact and the blisters were not aspirated.

**Figure 2:**
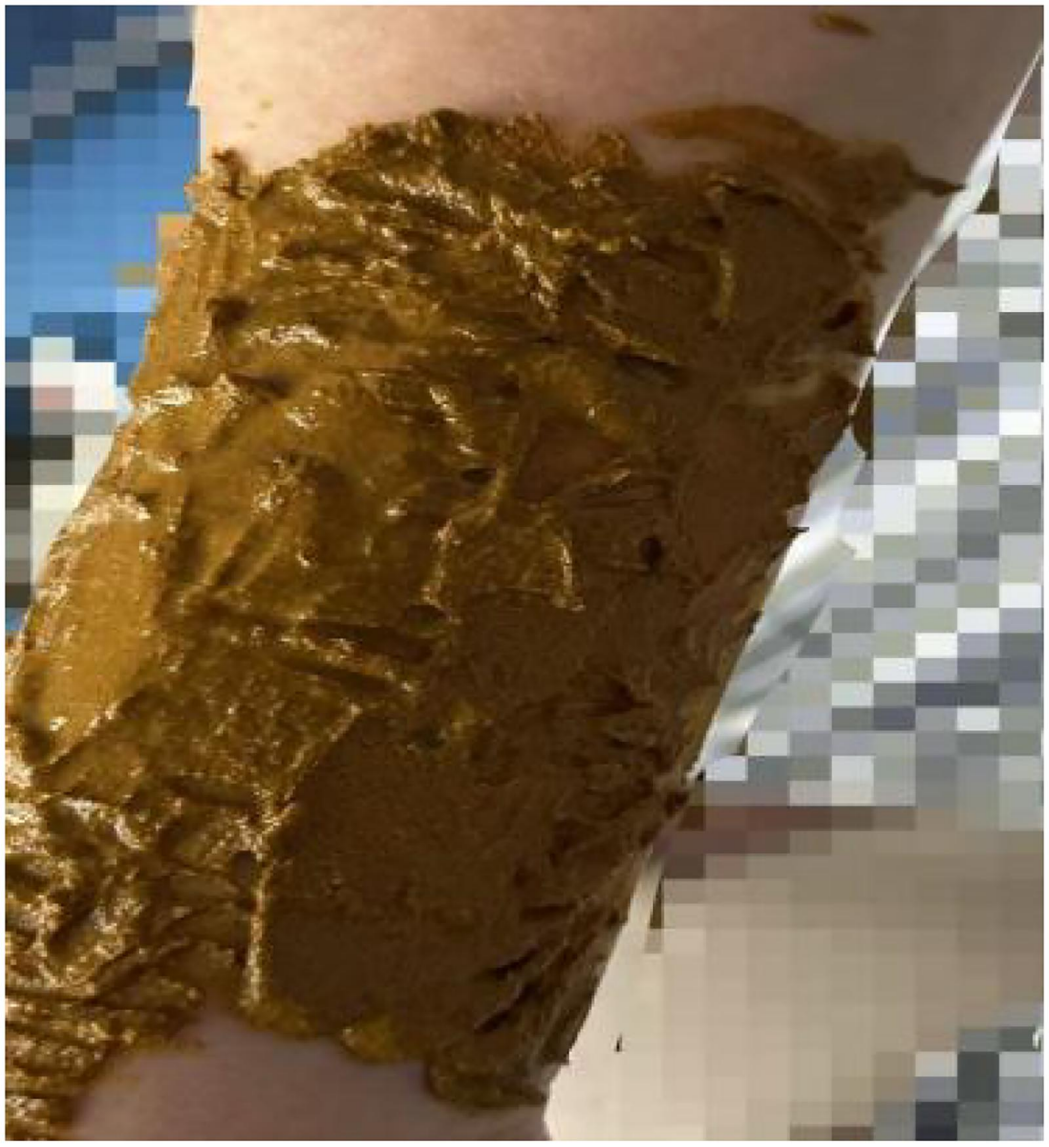
External application of Jinhuang San (golden yellow powder) mixed with green-tea water as a wet poultice over the affected forearm.

The differential diagnosis at the time of blistering included allergic/contact dermatitis to the antiseptic or dressing, skin infection, thermal injury, and primary vesiculobullous dermatoses; the close temporal relationship with the contrast injection, the strict confinement of lesions to the injection site, and the absence of pruritus, fever, or systemic symptoms supported extravasation injury as the cause.

On the following day (approximately 24 h after extravasation), significant improvement was observed: the blisters had begun to be absorbed, pain was markedly reduced, and the skin discoloration was fading (Figure 3). No skin breakdown, ulceration, or signs of infection were noted. Topical care was continued, and by 48 h after the event, the extravasation injury had resolved completely without further complications. From 22 January 2026, the NRS pain score was 0/10. At the departmental follow-up on 25 January 2026, the skin was found to be intact, without hyperpigmentation or functional deficit. The overall episode of care concluded on 19 February 2026. The complete clinical course is summarized in Table 1 and visualized in Figure 4.

**Figure 3:**
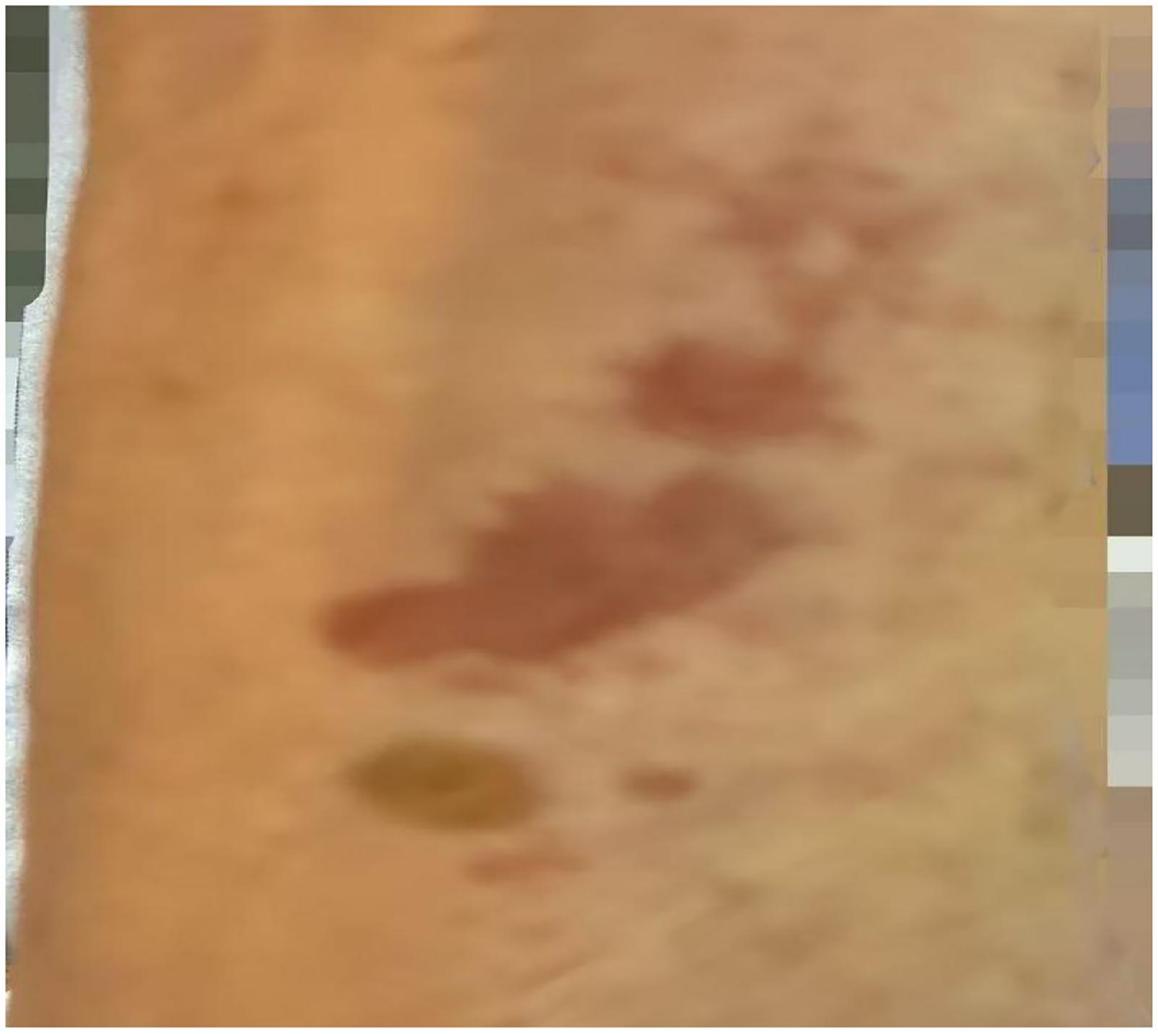
Approximately 24 h after the combined topical treatment regimen (Hirudoid cream, ice compress, and JinhuangSan tea-water poultice) was initiated, the skin blisters were resolved without any skin breakdown.

**Table 1:** Clinical timeline of contrast media extravasation, management, and outcome.

Date/time	Time since extravasation	Event/clinical finding	Management and outcome
7 years before admission	—	Onset of severe left lower-extremity pain, aggravated by standing/dependency, relieved by lying/elevation	No medical attention sought
1 year before admission	—	Symptom worsening with coldness of the left calf and foot	Outpatient evaluation; admitted with lower-extremity atherosclerotic occlusive disease
19 January 2026, 17:00	0 h	Contrast extravasation during lower-extremity CTA (iobitridol 350 mgI/mL, 80 mL at 3.5–4.0 mL/s via 20-G forearm cannula); distending pain at the puncture site (NRS 6/10)	Injection terminated immediately; cannula removed, site changed, CTA completed; extravasated volume ∼5 mL on bedside ultrasound
19 January 2026, evening (return to ward)	∼0.5–1 h	Pain around the puncture site; no obvious swelling	Hirudoid cream applied topically (thin layer)
19 January 2026, 22:00	∼5 h	3–5 tense fluid-filled blisters; affected skin ∼3 cm × 5 cm; erythema and mild swelling (Figure 1); NRS 4/10; no neurovascular compromise	Intermittent ice-bag compresses (15–20 min every 2–4 h, cloth barrier); Jinhuang San + green-tea-water poultice (1:1 paste, ∼2–3 mm, gauze-covered, changed q12h; 2 applications in total) (Figure 2); Hirudoid cream; limb elevation; hourly skin assessment
20 January 2026	∼24 h	Blisters being absorbed; pain markedly reduced; discoloration fading (Figure 3); no skin breakdown	Continued topical regimen
21 January 2026	∼48 h	Complete resolution of extravasation injury; intact skin	Continued observation
22 January 2026	∼72 h	NRS pain score 0/10	—
25 January 2026	6 days	Departmental follow-up: skin intact, no hyperpigmentation or functional deficit	—
19 February 2026	1 month	Overall episode of care concluded; no sequelae	—

**Figure 4:**
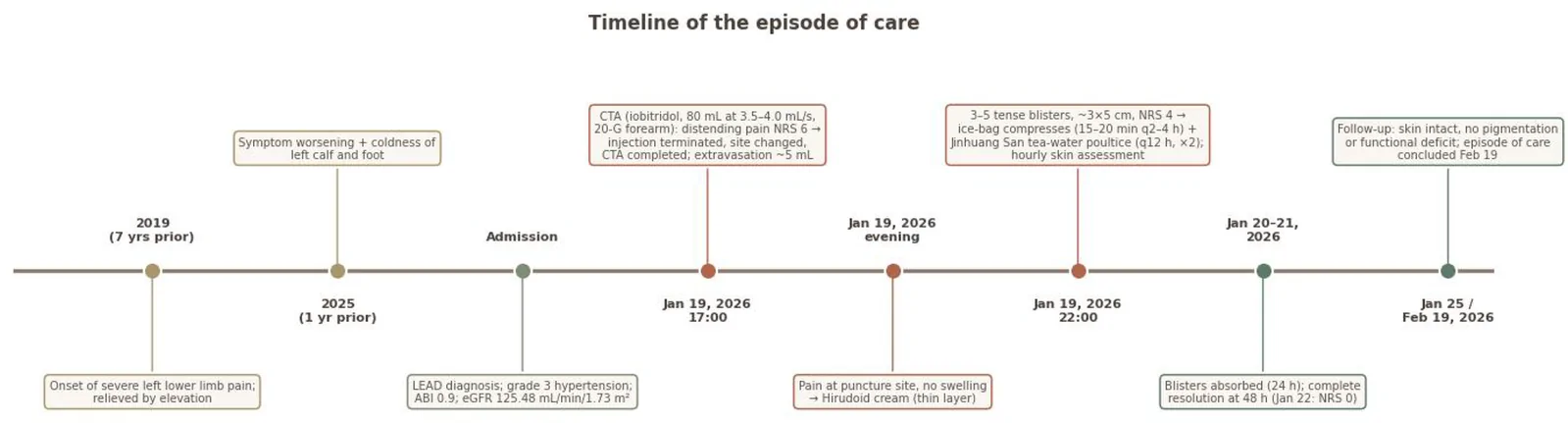
Graphical timeline of the extravasation event, symptom evolution, interventions, and outcome.

## 3 Discussion

### 3.1 CME and skin blistering: a common complication with an uncommon presentation

CME is an important complication of intravenous contrast administration during CT examinations, with a reported incidence rate of 0.1%–1.2% (2, 3). The large majority of events are mild and self-limiting; in a national data registry analysis, it was found that mild, moderate, and severe injuries accounted for 94.6%, 4.7%, and 0.8% of events, respectively (8). Blister formation is uncommon and tends to signal moderate-to-severe injury: in the cohort reported by Wang et al., 29 of 32 moderate extravasation injuries (90.6%) and all 5 severe injuries presented with blisters, with mean recovery times of 5 days (range 2–12) and 23 days (range 17–30), respectively (5). Against this background, the complete resolution achieved within 48 h in our patient—without skin breakdown—represents a favorable outcome at the milder end of the moderate-injury spectrum, plausibly facilitated by the relatively small extravasated volume (approximately 5 mL) and by prompt recognition and treatment.

### 3.2 Mechanisms of extravasation injury and the physicochemical properties of iobitridol

Three principal mechanisms are thought to contribute to contrast extravasation injury: (i) direct cytotoxicity of the contrast molecule toward endothelial and dermal cells; (ii) mechanical compression of tissues and microvessels by the extravasated fluid, raising interstitial pressure and compromising perfusion; and (iii) hyperosmolality-driven fluid shifts, which draw water into the interstitial space, producing edema, cellular dehydration, and further elevation of tissue pressure (7). Classic experimental work demonstrated that contrast extravasation can produce a dose-dependent inflammatory and ulcerative cutaneous response (11).

Iobitridol is a non-ionic, low-osmolar monomer, yet at the concentration used for CTA (350 mgI/mL), its osmolality is approximately 915 mOsm/kg—roughly three times that of plasma—with a viscosity of approximately 10.0 mPa·s at 37 °C (12). Although non-ionic low-osmolar agents are substantially safer than ionic high-osmolar media (5, 6, 13), a hypertonic depot of extravasated iobitridol can still provoke osmotic edema, microvascular irritation, and sterile inflammation, which in sequence may separate the epidermis from the dermis and produce the serous blisters observed in our patient. *In vitro* comparative data confirm that iobitridol exhibits cytotoxic and hemorheological effects qualitatively similar to other non-ionic monomers (12, 14). Our patient's underlying peripheral arterial disease may additionally have impaired local tissue perfusion and venous integrity, potentially lowering the threshold for clinically evident injury, although this remains speculative.

### 3.3 Rationale and mechanisms of the combined topical regimen

#### 3.3.1 Mucopolysaccharide polysulfate (Hirudoid)

MPS is a heparinoid with anticoagulant, anti-inflammatory, and antiedematous properties that improve local microcirculation when applied topically (15). In superficial thrombophlebitis—a condition sharing the features of perivascular inflammation, pain, and edema—topical heparinoids are supported by randomized-trial evidence summarized in a Cochrane review (16), and in one comparative study, it was found that topical MPS shortened symptom resolution from 126 to 58 h (*P* < 0.05) (15). Beyond vascular effects, a topical heparinoid-containing product has been shown to restore epidermal permeability-barrier homeostasis by upregulating filaggrin and related barrier proteins, providing a plausible mechanistic link to accelerated blister re-epithelialization (17).

#### 3.3.2 Jinhuang San (golden yellow powder/Ru-Yi-Jin-Huang-San)

This classical external formula contains Trichosanthis Radix, Phellodendri Chinensis Cortex, Rhei Radix et Rhizoma, Angelicae Dahuricae Radix, Curcumae Rhizoma, and other herbs; component analyses have identified quercetin, berberine, emodin, and β-sitosterol as major active constituents with anti-inflammatory and antioxidant activities (18). Pharmacological studies show that the formula accelerates wound healing by modulating Wnt/β-catenin signaling in fibroblasts and downregulating proinflammatory cytokines such as IL-1α, IL-1β, and IL-6 (19); a retrospective cohort in acute gouty arthritis further supports its anti-inflammatory and analgesic clinical efficacy (20). Of direct relevance, in a rabbit iohexol extravasation model, it was found that Jinhuang San ointment significantly reduced swelling area and inflammatory infiltration at 24 and 48 h, respectively (*P* < 0.01) (10), and early wet compresses with the formula achieved complete healing of drug extravasation skin injuries in a mean of 6.44 days in clinical use (9). The green-tea-water vehicle is itself mildly astringent and rich in catechins; the cool, moist poultice additionally provides a soothing, evaporative-cooling effect on the inflamed skin.

#### 3.3.3 Guideline support

The combination used here is consistent with the Chinese Nursing Association practice guideline for nursing management of intravenous extravasation of iodinated contrast media, which recommends limb elevation plus cold compresses for mild extravasation (Grade A recommendation) and topical magnesium sulfate, mucopolysaccharide ointment, or Ru-Yi-Jin-Huang-San application for moderate-to-severe extravasation (Grade B recommendation) (21). Our case, with blistering consistent with moderate injury, therefore maps directly onto this grade-B-supported topical pathway. General extravasation guidelines similarly endorse conservative, symptom-directed topical care with structured monitoring as first-line management (1, 4, 22).

#### 3.3.4 Cold vs. heat

The optimal thermal modality remains debated. A multicenter trial found that dry-heat compresses applied within the first hour achieved complete resolution in 90.9% of patients vs. 40.6% with cold compresses (23). In our case, ice compresses were chosen to limit early hyperosmolar edema and to provide analgesia, and recovery was nonetheless rapid; whether heat would have further accelerated resolution cannot be determined from a single case, and this question merits a controlled study.

### 3.4 Comparison with similar published cases

Published case reports of CME with blistering have predominantly described severe injuries with poor outcomes despite, or following the failure of, conservative care (Table 2). Hu et al. reported a 53-year-old woman with a 75-mL iohexol extravasation in whom conservative management (elevation, cold compresses, mucopolysaccharide polysulfate, magnesium sulfate, and dexamethasone) failed, with progression to blistering, necrosis, and ulceration requiring debridement on day 10 and healing with scarring by day 60 (24). Diop et al. described a 47-year-old woman in whom extravasation of 100 mL of a high-osmolality agent into the dorsum of the hand led within 24 h to blistering, necrosis, and compartment syndrome requiring fasciotomy, with recovery over 2 months (25). Belzunegui et al. reported a 50-year-old woman who developed hand compartment syndrome with blistering after CT contrast extravasation, also requiring fasciotomy (26). In contrast, our patient presented with blistering but no neurovascular compromise, received an integrated topical regimen within hours, and recovered completely within 48 h. The contrast underscores two points: first, outcome in CME correlates strongly with extravasated volume, agent osmolality, and site (the hand being particularly vulnerable to compartment syndrome); second, early, protocolized topical management may help arrest progression at the blistering stage, although the single-case design precludes causal inference.

**Table 2:** Comparison with published cases of contrast media extravasation presenting with skin blistering.

Study	Age/sex	Contrast agent and volume	Site	Manifestation	Treatment	Outcome/recovery time
Present case	68/F	Iobitridol 350 mgI/mL; ∼5 mL extravasated of 80 mL administered	Forearm	Distending pain during injection (NRS 6/10); 3–5 serous blisters at ∼5 h (NRS 4/10); no neurovascular compromise	Hirudoid cream + ice compresses + Jinhuang San tea-water poultice + elevation	Complete resolution in 48 h; no skin breakdown
Hu et al. 2025 (24)	53/F	Iohexol, 75 mL extravasated	Upper extremity	Blistering → necrosis and ulceration despite conservative care	Elevation, cold compress, MPS, magnesium sulfate, dexamethasone; debridement on day 10	Healed with scarring by day 60
Diop et al. 2022 (25)	47/F	High-osmolality agent, 100 mL	Dorsum of hand	Blisters and necrosis within 24 h; compartment syndrome	Emergency fasciotomy	Recovery over ∼2 months
Belzunegui et al. 2011 (26)	50/F	Iodinated CT contrast (volume not reported)	Dorsum of hand	Blistering; hand compartment syndrome	Emergency fasciotomy	Recovered after surgery

### 3.5 Advantages of the proposed regimen

The combined regimen has several practical advantages. All components are topical, inexpensive, widely available, and easily administered at the bedside by nursing staff without specialized equipment. The components act on complementary targets—MPS on microcirculation, inflammation, and epidermal barrier repair (15–17); ice on acute edema and pain; and Jinhuang San on cytokine-mediated inflammation and wound repair (18–20)—providing a biologically plausible basis for synergy. The regimen is consistent with national nursing guidelines (21), is well tolerated, and, in the case of our patient, was temporally associated with blister absorption within 24 h and complete resolution within 48 h—substantially shorter than the mean 5-day recovery reported for moderate injuries with blistering in cohort data (5). It may therefore represent a pragmatic option for early, moderate CME with intact skin, particularly where rapid escalation to surgery is not indicated.

### 3.6 Limitations

Several limitations in this study must be emphasized. First, this is a single-case study without a control group; the favorable outcome may reflect the natural history of a relatively small-volume extravasation rather than treatment efficacy, and no causal attribution to any component of the regimen is possible. Second, the proposed mechanisms of both injury and treatment are inferred from experimental and indirect clinical literature; no histological, biochemical, or imaging confirmation of the postulated pathways was obtained in our patient. Third, key quantitative details (extravasated volume, blister dimensions, pain scores) were abstracted only from the injector record and nursing documentation, and therefore, minor documentation gaps inherent to retrospective case reporting may remain. Fourth, the evidence base for Jinhuang San derives largely from Chinese-language studies, animal models, and small uncontrolled cohorts, limiting external validity. Fifth, our choice of ice compresses conflicts with trial data favoring dry heat (23); the optimal thermal strategy is unresolved. Finally, blistering can herald delayed deterioration: compartment syndrome has been reported to evolve within hours after an initially benign-appearing extravasation managed with elevation and ice (27). Vigilant serial neurovascular monitoring—and a low threshold for surgical consultation—therefore remains mandatory even when topical therapy appears successful.

## 4 Conclusion

Skin blistering is an uncommon, generally moderate-severity manifestation of contrast media extravasation. In this single case, iobitridol extravasation during lower-extremity CTA presented with delayed blistering approximately 5 h after injection and resolved completely within 48 h during combined topical treatment with Hirudoid cream, ice compresses, and a Jinhuang San tea-water poultice. As a single-case observation, this report is hypothesis-generating only: the regimen appears to have been safe and may have contributed to rapid resolution, but efficacy cannot be established at this level of evidence. This case supports the value of early recognition, guideline-concordant topical care, and structured monitoring and suggests that integrating traditional Chinese medicine external therapy with conventional agents merits evaluation in controlled, adequately powered studies. Clinicians should remain vigilant for this complication, particularly in patients with underlying vascular disease, and preventive measures—including appropriate cannula selection, saline test injection, and power-injector monitoring—should be strictly followed during contrast-enhanced CT procedures (1, 3, 4).

## Data Availability

The original contributions presented in the study are included in the article/Supplementary Material; further inquiries can be directed to the corresponding author.
